# A Comprehensive Overview of Antibiotic Selection and the Factors Affecting It

**DOI:** 10.7759/cureus.13925

**Published:** 2021-03-16

**Authors:** Karan Patel, Sean Bunachita, Ank A Agarwal, Akshay Bhamidipati, Urvish K Patel

**Affiliations:** 1 Medicine, Cooper Medical School, Camden, USA; 2 Molecular and Cellular Biology, Johns Hopkins University, Baltimore, USA; 3 Medical Education, Johns Hopkins University, Baltimore, USA; 4 Medicine, Vanderbilt University Medical Center, Nashville, USA; 5 Public Health and Neurology, Icahn School of Medicine at Mount Sinai, New York, USA

**Keywords:** clinical decision making, antibiotic selection

## Abstract

In order to prescribe an antibiotic, a physician must go through a series of decision-making processes that involve both the drug and the host. In this review article, we outline exactly what those decision-making processes are and some of their limitations. Before a medication can be prescribed, a physician has to determine if the antibiotic works against the host pathogen. To do this, basic science techniques are employed including phenotypic methods such as broth dilution methods, Kirby-Bauer susceptibility testing, Epsilometer test (E-test), and genotypic methods such as the new and upcoming automated tests. After determining if a drug has potential to work, the physician must consider the drug’s mechanism of action in order to determine a dosing regimen. Some groups of drugs should be administered at high concentrations infrequently, others should be given more frequently in smaller doses, and others lie somewhere between this spectrum. Finally, external factors such as the patient's age, especially for pediatrics and geriatrics patients, need to be considered, as these groups have the highest health care burden but are among the most vulnerable when it comes to the side effects of drugs.

## Introduction and background

Pharmacokinetics, in a layman's terms, has been described as the effect the body has on the drug, while pharmacodynamics has been described as the effect of the drug on the body [1]. These two factors are perhaps the most important factors to consider when a physician or any health care professional prescribes medication to an individual. Pharmacokinetics is generally broken down into four major categories: Absorption, Distribution, Metabolism, and Excretion, widely referred to as ADME. Absorption refers to the transport of a drug from its site of administration to the site of measurement [2]. There are multiple routes of administration, such as oral or intravenous (IV), and each can affect bioavailability (the amount and rate of active drug that reaches the bloodstream). For example, more drugs are going to be available through an IV route compared to an oral route [3]. Distribution refers to where in the body the drug ultimately ends up [4]. Drugs that are more lipophilic remain in tissues while drugs that are more hydrophilic tend to stay in the bloodstream [3]. Metabolism refers to the breakdown of drugs and their conversion into a different compounds after entering the body [2]. The primary method through which this is done is hepatic phase 1 and phase 2 enzymes that generally increase the polarity of drugs (phase 1 by modifying pre-existing structures on drugs) and phase 2 (by adding polar structures) [5]. Finally, excretion refers to the elimination of the drug from the body that is generally handled by the renal system [4]. The pharmacodynamics of a drug is based on three main principles: (1) the amount of drug that binds to receptors, (2) the ability of the drug to influence receptors, and (3) the amount of time the drug is present so that it can exert its metabolic effect [6]. While pharmacokinetic principles are absolutely necessary in the development of the drug, the pharmacodynamic effects of the drug are what clinicians focus on. Although these are the main considerations of many physicians while prescribing medications, there are also additional factors to consider, such as which pathogens will be susceptible to a particular drug. The physician must also account for host factors including age (pediatric versus geriatric), polypharmacy, and organ dysfunction as those play a major role in determining which drugs are appropriate [7].

## Review

Before even considering how a patient will both affect and be affected by a drug, a physician must determine exactly which pathogen is responsible for the disease and if a drug will be effective in treating a disease. Pathogen identification and antibiotic susceptibility are two distinct phases and require vastly different techniques. Pathogen identification uses either older phenotypic techniques such as various agar plates that are selective (only allow certain bacteria to grow) and differential (bacteria with different properties appear differently on the agar plate) or newer genotypic methods including DNA or RNA sequencing [8,9]. For the purposes of this review we primarily focus on the latter, including antibiotic susceptibility, mechanisms of antibiotics, pharmacokinetics and pharmacodynamics of medications, and other factors to consider when prescribing medications.

Although historical clinical decision making generally followed a binary process where condition X was treated by drug Y, the rampant use of antibiotics has now added another factor to this already complex decision-making process. New strains of antibiotic-resistant pathogens are constantly emerging [10]. In order to determine if a pathogen is susceptible to an antibiotic, the pathogen has to be sent to the laboratory, where basic science techniques are applied. In the following, we review the four most widely used methods to determine antibiotic susceptibility and discuss some of their limitations.

Methods to determine pathogen susceptibility

Broth Dilution

During broth dilution, various concentrations of antibiotics are prepared in test tubes. The first test tube has the highest concentration of antibiotic and each test tube thereafter presents with a twofold dilution of the antibiotic in the prior test tube [11]. For example, if the first test tube had a concentration of 8 mg/ml, the next test tube is prepared with an antibiotic concentration of 4 mg/ml, the next tube has 2 mg/ml, and so forth. In order to test multiple concentrations and multiple antibiotics at once, these dilutions can be done in a 96-well plate. Afterward, the bacteria are added to the plates and then inoculated for approximately 20 hours. This method relies on observational principles: if the bacteria grow, they cause the solution they are in to become cloudy and turbid; if there is no bacterial growth, then the solution will remain clear [11]. The concentration at which the antibiotic is effective is then determined by the researcher based on which concentration of the antibiotic produced a clear solution, known as the minimum inhibitory concentration (MIC) [12]. This concentration is then compared to a table produced by the Clinical Laboratory Standards Institute (CLSI) to determine if a bacterium is susceptible, intermediate, or resistant to the antibiotic [11]. This method also allows researchers to determine the lowest concentration of an antibiotic that is able to eliminate 99.9% bacteria, also known as the minimum bacterial concentration (MBC) [13]. The test tubes or wells can be viewed under a microscope to determine this concentration.

Kirby-Bauer Test

**Figure 1:**
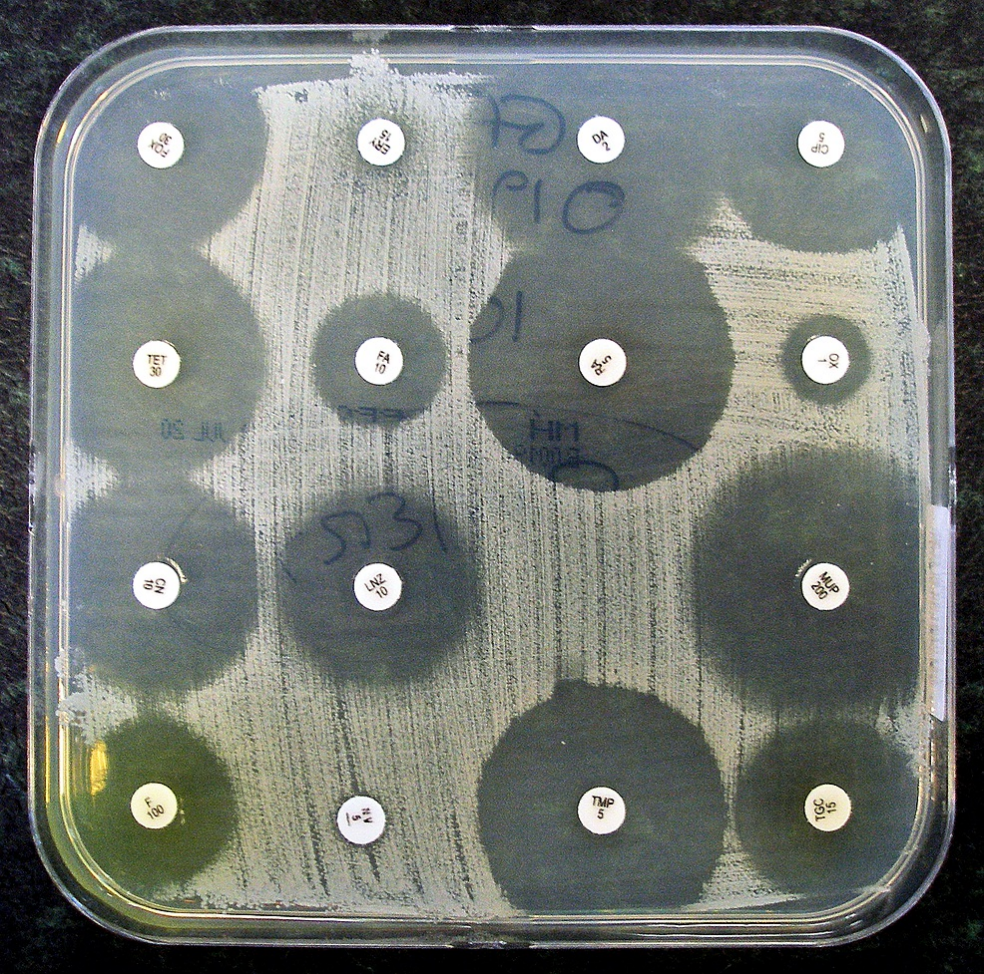
Kirby-Bauer disk diffusion test Original image by Dr. Graham Beards, distributed under a CC BY-SA 4.0 license. No modifications were made. Source: [17]

The Kirby-Bauer disk diffusion method (Figure 1) is a qualitative method that allows a researcher to test the effect of multiple drugs on a plate at once [14]. Essentially, the pathogen of interest is placed on a Mueller-Hinton plate and streaked throughout the plate [15]. Then, various small and circular filter disks with high concentrations of antibiotics are arranged throughout the plate [15]. This method relies on diffusion principles: the small circular concentrates of antibiotics will spread across the plate, with the region closest to the circular antibiotic filter containing the highest concentrations [14]. After incubating the bacteria overnight, or as long as needed (depending on the bacteria of interest), there will be circular zones (known as zones of inhibition) around the antibiotic filters. These circular zones are then measured and compared to a set of data put forth by the CLSI. Depending on the size of the zone of inhibition, the region around the circular antibiotic filter where bacteria did not grow, the antibiotic will be classified in one of three categories: resistant, susceptible or intermediate [15]. One of the primary limitations to this method is that it is purely qualitative in nature, though some commercial products can calculate approximate MICs by comparing zone of inhibition sizes to those of a standardized curve [16].

E-Test

**Figure 2:**
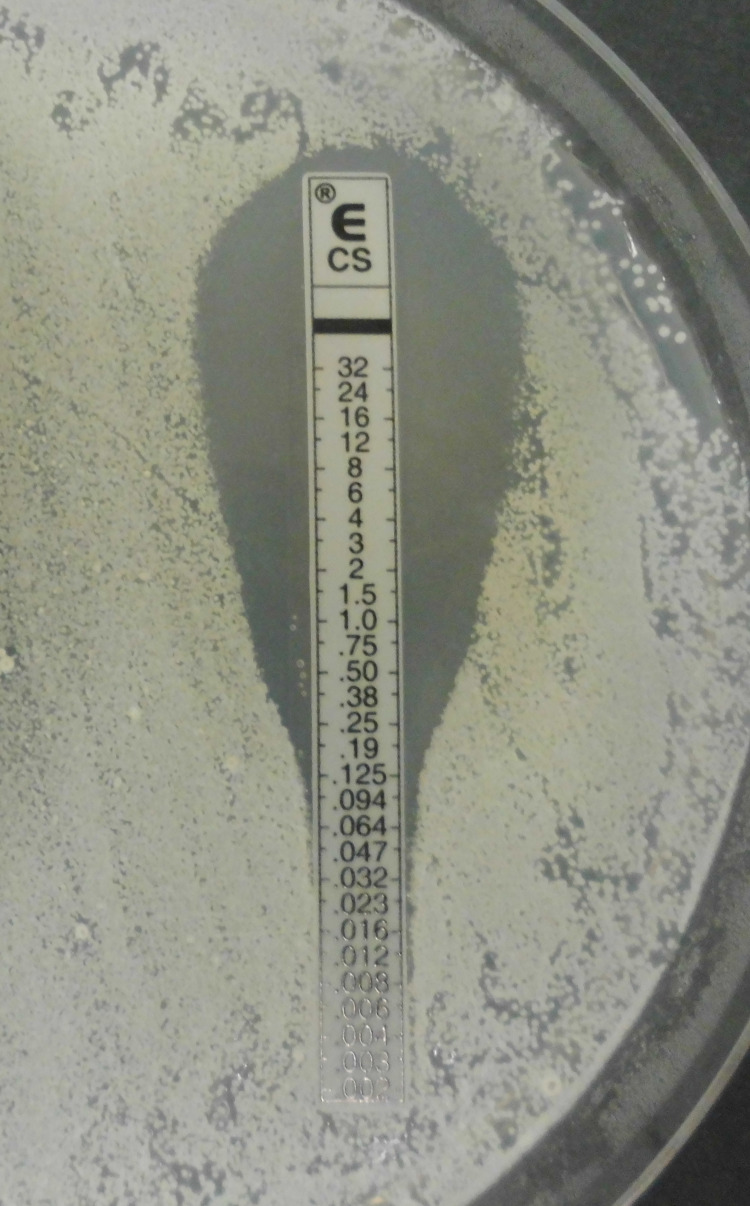
E-test Original image by Wikipedia user *Garnhami* distributed under a CC BY-SA 4.0 license. No modifications were made. Source: [21]

An Epsilometer test (E-test) combines the principles of a broth dilution and Kirby-Bauer test in order to determine the MIC of a drug. An E-test is performed with an antibiotic strip that has a continuous concentration gradient of the drug along the strip [18]. For example, the top of the strip may contain a drug concentration of 10 mg/ml while the bottom of the strip contains a concentration of 2 mg/ml, with varying increments of concentrations contained throughout the middle. Similar to a Kirby-Bauer test, bacteria are streaked on a plate, the strip is placed on the plate, and the plate is incubated overnight. Because of the varying concentrations of the antibiotic across the strip, there will be various sizes of zones of inhibition along the strip [19]. The concentration at which the smallest possible zone of inhibition forms is the MIC [18]. While this method is extremely time consuming and labor intensive, it allows researchers to determine which bacteria may acquire new resistance mutations [18]. For example, in a 2005 study, an E-test was markedly more accurate at detecting carbapenem-resistant *Klebsiella pneumoniae* compared to automated susceptibility testing [20]. Figure 2 shows an example of the results of an E-test.

Automated Systems

Currently, we are finding ourselves in a transition period between using classic laboratory techniques to determine susceptibility and using automated machines that can expedite and aid us in providing a diagnosis. Machines such as GeneXpert® (Cepheid, Sunnyvale, CA) have begun to be employed that allow for not only the determination of a pathogen, but also the determination of resistance to certain antibiotics such as methicillin - a popular use of the machine is to help diagnose methicillin-resistant *Staphylococcus aureus* (MRSA) [22,23]. Moreover, BioFire machines (BioFire Diagnostics, Salt Lake City, UT) can identify pathogens directly from blood cultures and can detect resistance to methicillin, vancomycin, and carbapenem [24]. Despite historically being considered expensive to implement, low-cost systems are growing in popularity. Still, many hospitals must do a cost-benefit analysis before purchasing these machines. Questions that are considered in the decision making include the following: (1) Will there be people available to treat the patient faster if the diagnosis is given earlier? (2) Does it improve patient outcomes? (3) Will it save money in the long term due requiring fewer laboratory tests to help figure out a diagnosis? Also, as mentioned earlier, another downside is that these machines may not be able to catch novel mutations in pathogens [20].

Susceptibility Reports

Susceptibility reports are essentially the results of the basic science testing described above, which are given to the physician to guide them to the best course of treatment. These generally include the name of the pathogen, the drugs tested on the pathogen, and whether the pathogen is susceptible, intermediate, or resistant to the drug. Although the meanings behind the three outcomes are self-explanatory, there is some nuance to these distinctions. Susceptibility generally indicates a favorable clinical outcome if the treatment course is followed, and perhaps most importantly, suggests that normal amounts of the drug will allow for serum levels to be well above the MIC [25]. A result of intermediate suggests that the drug may or may not be effective depending on the following: (1) the drug needs to be given at high doses in order to get serum levels above the MIC or (2) the drug may be effective if it concentrates at the site of infection [26]. Resistant predicts a poor clinical outcome and says that even maximal drug concentrations will not allow for serum levels to exceed the MIC [25].

Limitations to These Methods

While these in vitro methods do provide physicians with relevant clinical data, they do have serious limitations. For one, they do not account for a real-world time course of drugs in that they do not take into account dosing intervals or pharmacokinetic and pharmacodynamic drug changes. For example, in vitro testing methods assume that a concentration of the drug stays the same throughout the dosing interval, without accounting for decreases in concentrations and the half-life of drugs [27]. Moreover, these methods are not able to consider external effects on drugs including, but not limited to, drug-drug interactions as a result of polypharmacy, age-related organ decline, and pharmacokinetic variations in a population [7]. However, recent models such as the Monte Carlo system have begun to address some of these concerns. The Monte Carlo system is a computer-based model that integrates variables such as tissue distribution, antimicrobial susceptibility, and pharmacokinetics in a population to give a probability of how likely a drug is to achieve a target concentration in a population [28].

Pharmacodynamic and pharmacokinetic applications

After determining if a drug is going to be effective from a clinical standpoint, the physician must also consider other drug factors including dosing intervals. Various classes of drugs require different dosing times based on the ideal optimization of the drug. Ideally, drugs with longer dosing intervals are chosen for the patient’s convenience. However, this is not always possible because some drug classes are more effective with more frequent dosing intervals. In the next paragraph, we talk about the three primary mechanisms through which drugs operate.

Drug Mechanism

The first class of drugs are those that should be dosed in order to maximize their time spent above the MIC [29]. Drugs in these categories do not generally operate in a concentration-dependent manner, but rather have the same amount of effectiveness at any serum concentrations above the MIC and thus should be given in smaller, more frequent doses in order to maximize their effect [30]. Although a wide array of antibiotics fall within this category, the most prominent are the beta-lactams that include penicillin, cephalosporin, carbamazepine, and monobactams. Generally speaking, for these drugs to be effective, 50%-70% of the time spent between dosing intervals should be above the MIC [31]. The next class are drugs in which the peak concentration above the MIC should be maximized, rather than the time spent above the MIC [30]. A larger infusion of these drugs, particularly the aminoglycosides, will lead to more bacterial killing. A simulated study performed in the Hartford Hospital showed that there was increased effectiveness, and still minimal toxicity, in less frequent but larger doses while administering aminoglycosides [32]. Finally, the third most prominent category is between the first two where the ratio of the area under the curve (AUC) and the MIC (AUC/MIC) should be maximized [30]. In simpler terms, this third class of drugs is most effective when maximizing a patient's overall exposure to the drug. These three categories are not as set in stone or distinct as presented here. There is plenty of overlap between categories and many drugs such as vancomycin or clindamycin fall into multiple categories.

Many of the drugs that we currently use today have a wide therapeutic index (range of concentrations at which they are effective). However, some drugs require constant monitoring of therapeutic levels in order to make sure that their concentration falls within a therapeutic window. For example, aminoglycosides, vancomycin, and gentamicin all require constant serum monitoring, which can be an inconvenience to the patient [33,34]. While there are numerous other categories of drugs that fall into this category, we presented these as examples of some commonly prescribed antibiotics. If at all possible, the physician should consider drugs with a wide therapeutic index in order to avoid serum concentration monitoring for the sake of the patient. However, in certain cases this is not always possible to avoid as some conditions can only be treated by medications with narrow therapeutic windows.

Other factors to consider

Pediatric Populations

One of the challenges with pediatric populations is that although they have a high burden of disease, they are often excluded from clinical trials, making the prescription of novel medications difficult. This can be seen through the catastrophe that resulted from prescribing benzyl alcohol to pediatric patients even though it had cleared clinical trials. In adults, a preservative used in benzyl alcohol was harmlessly metabolized to hippuric acid, but in children that same preservative was metabolized to benzoic acid, causing respiratory distress and cardiovascular collapse [35]. Aside from the challenges associated with prescribing novel medications to pediatric patients, physicians also have to account for their increased intracellular volume, resulting in higher volume of distribution and reduced plasma binding proteins. Pediatric doses cannot simply be extrapolated based on surface area (Clark's rule), height, and weight (Hong's rule) from adult clinical trials [36]. The danger of this can be seen in dosing lipophilic drugs, such as diazepam, to children based on adult doses [37]. Because pediatric patients do not have the same levels of plasma binding proteins, more of a drug than expected will be in its active form, resulting in overdoses. Moreover, children have immature hepatic and renal enzymes that do not allow them to metabolize and excrete drugs as efficiently as adults [37].

Geriatric Populations

Geriatric populations also have many of the same complications as pediatric populations. They too are widely affected by diseases but are often excluded from clinical trials. The primary challenges with geriatric patients include physicians having to account for a decline in organ function and polypharmacy [38]. Studies have typically shown that after 30 years of age, organ functions decline by 1% percent per year (with interindividual variability) [39]. As a result, the physician must account for the decline in absorption, metabolism, and excretion when prescribing medication [38]. Furthermore, the distribution of medication is also affected, because as age increases, the ratio of intracellular to extracellular volume decreases [40]. Polypharmacy is arguably the most complicated factor. As the disease burden increases with age, so too does the number of medications. Many medications have the ability to either upregulate or downregulate hepatic metabolic enzymes such as the CYP450 enzymes, which may affect the concentrations of other concurrently administered medications [41]. For example, common prescribed drugs such as macrolides and azoles have the ability to inhibit CYP450 enzymes, leading to increased concentrations of other drugs, while drugs such as rifampicin and rifabutin may induce CYP450 enzymes, leading to the decreased concentration of other drugs [42]. Medications that have a narrow therapeutic window may either exceed their concentrations leading to toxicity (if hepatic enzymes, primarily CYP450s, are downregulated) or may not achieve a concentration in the therapeutic window (if CYP enzymes are upregulated) [41].

Pregnant Populations

Pregnancy is another important factor for physicians to consider when prescribing antibiotics. Pregnant women undergo physiological changes that have various consequences for drug levels and effects. While each drug may be affected in unique ways, the general effects of pregnancy include slower absorption as a result of decreased gastric emptying, increased plasma volume leading to a greater volume of distribution for lipophilic drugs, an increased glomerular filtration rate (GFR) leading to an increase in drug elimination, and decreased plasma protein concentrations [43,44]. These can be difficult to manage since many of the changes during pregnancy have clashing effects. For example, decreased plasma protein concentrations lead to an increase in the level of active drugs, but an increase in GFR decreases the overall time a drug is active since it will be excreted faster. Beyond these concerns, the effect of drugs on the fetus has to be of utmost consideration. For instance, antibiotics with a pregnancy category of D, such as doxycycline, should be avoided at all costs as they are known to cause birth defects or complications [45]. The physician must play a delicate balancing act between prescribing sufficient doses of a drug to ensure it is active, but not so high of a dose such that it may have adverse effects to the patient and the fetus.

Routes of Administration

The physician must keep in mind the convenience of the patients. While certain IV-administered medications may be superior to a different oral medication, there has to be a cost-benefit analysis when prescribing the IV medication. IV medications are more difficult for patients to administer and many times may not be able to be used in an outpatient setting [46]. Outpatient Parenteral Antibiotic Therapy (OPAT) programs have been designed in order to address some of these challenges. These programs were created in order to administer IV antibiotics outside of hospital care settings. In these programs, a nurse or other healthcare provider conducts an initial visit in which they instruct the patient or caregiver on how to administer medication in an outpatient setting. The healthcare professional may return for follow-up visits to perform tasks such as drawing blood for labs or redressing wounds [47]. However, even though OPAT programs allow for the administration of IV drugs in an outpatient setting, they still add additional challenges to patient care. Therefore, if two medications are of relatively equal effectiveness, physicians often will prescribe the oral formulation due to convenience. Unfortunately, there do present situations where IVs are necessary as they typically have higher bioavailability and deeper penetration of tissues [48]. While there are other routes of administration, we focused on oral and IV as those are the most common routes of intake.

Antimicrobial Stewardship

Much of the issue of contemporary drug-resistant bacteria can be attributed to the over-prescription of antibiotics in past decades. Constant exposure to antibiotics leads to selective pressures that allow for bacteria to evolve and develop resistance to the drugs. In order to help combat this, the Centers for Disease Control and Prevention (CDC) implemented the Antimicrobial Stewardship (AMS) program in 2014. The main purpose of AMS is threefold: (1) preventing the misuse of antibiotics (2) minimizing the resistance to antibiotics (3) having each patient receive appropriate drug doses. In order to maximize these goals, each hospital works within their own parameters to create teams, which typically consist of infectious disease physicians and pharmacists who then collaborate with microbiology staff in order to formulate best practices. Two main approaches have been effectively employed. The first approach is to create a restrictive prescriptive authority, in which certain medications can only be prescribed after consultation with members of the AMS team. The other approach consists of a review system, where the AMS team reviews antibiotic orders and then provides the physician with recommendations for the future [49]. As of 2018, 85% of hospitals in the United States are meeting the major core goals of the AMS program. The widespread implementation of AMS has led to more effective antibiotic use and improved patient outcomes [50].

## Conclusions

Various factors go into clinical decision making when a physician prescribes a medication. These decisions include determining susceptibility, dosing regimens, and considering external factors such as age-related effects and routes of administration. Whereas historically, a patient's disease was viewed more in a binary sense (disease X was treated by drug Y), progress in medicine has shown us that much more needs to be considered. The initial, oversimplified model has led to numerous unfortunate events such as lethal benzyl alcohol incidents in pediatric patients, cases of adverse polypharmacy interactions in geriatric patients, and inappropriate dosing in pregnant patients. The over-prescription of antibiotics has added another layer of complication. Organisms have developed new mechanisms to evade the drugs, causing susceptibility testing to become an ever more important factor in clinical decision making. While we are currently making progress in order to combat drug resistance through AMS programs, physicians today face far greater challenges than in the past. Today, they must consider a wider range of variables when administering medications.
